# Incidence and Outcomes of Transplant of Infected Donor Corneal Tissues in a Tertiary Hospital in Saudi Arabia

**DOI:** 10.7759/cureus.25514

**Published:** 2022-05-31

**Authors:** Ammar Almulhim, Abdullah F Alnaim, Ahmed Abdulrazek, Hammam A Alotaibi

**Affiliations:** 1 Ophthalmology, Al-Jabr Eye and ENT Hospital, Al-Ahsa, SAU; 2 Ophthalmology, Dhahran Eye Specialist Hospital, Dhahran, SAU; 3 Research, Prince Sultan Military Medical City, Riyadh, SAU

**Keywords:** keratoplasty, corneal graft rejection, graft contamination, infection, corneal transplantation

## Abstract

Purpose: The aim of this study is to assess donor rim culture results and predict ocular infections after corneal transplantation to determine the relationship between positive corneoscleral rim cultures and post-keratoplasty infection.

Design: This is a retrospective study.

Methods: The microbiology results of positive donor rim culture and clinical outcomes of all contaminated grafts at Dhahran Eye Specialist Hospital (DESH) in the Eastern Region of Saudi Arabia between October 2016 and February 2020 were reviewed.

Results: A total of 684 corneal transplantation procedures were performed at DESH during this period. Routine donor rim cultures were done for all cases with six positive cases (0.88%). There were five positive fungal donor rim cultures (0.73%), and one corneal rim was positive for both bacterial and fungal cultures (0.15%). Among these six patients, two patients (33.33%) who received graft positive with candida developed an infection. Both cases required further interventions but were unsuccessful and eventually ended up with re-grafting.

Conclusion: In the sample collected, positive fungal contamination was more frequent than bacterial contamination, and the incidence of bacterial infection is relatively low compared to that of fungal infection due to contaminated grafts. Despite the limitations of our study, we support routine corneoscleral rim culture and to start prophylactic antifungal treatment in response to positive donor fungal rim culture.

## Introduction

Keratitis or endophthalmitis post-keratoplasty due to contaminated donor corneal tissue is a rare but serious complication that may lead to significant visual loss and can even finally result in evisceration. Although perioperative culturing of the corneal rim is aimed to minimize the risk of postoperative infections, there are no specific perioperative protocols and standards in the medical literature for culturing the corneal rim, and some reports claimed that such testing has no value in predicting infectious complications post-keratoplasty [1,2]. Nowadays, there are two basic approaches to preserve corneas for grafting: hypothermic and organ culture methods [3].

Hypothermic storage at 2°C-8°C is the method of choice for corneal preservation in North America and about 30% of member banks of the European Eye Bank Association (EEBA). Optisol-GS (Bausch & Lomb, Rochester, NY) is the most extensively used commercially available medium for hypothermic storage, which contains 2.5% chondroitin sulfate, 1% dextran, ascorbic acid, vitamin B12, adenosine triphosphate precursors, gentamicin, and streptomycin. The recommended storage time under hypothermic conditions is 14 days. Before corneal tissue is issued for transplantation, a slit lamp and specular microscopy examination are performed to assess the endothelial cell density and tissue quality. On the day of surgery, the cornea is trephined in the operating room, and the corneal rim is then sent for a microbiology workup. The main advantages of hypothermic storage are faster, immediate availability of tissue and reduced cost compared to the organ culture method [3].

Organ culture (OC) storage techniques are the most commonly used method in Europe. Minimum essential medium (MEM) is the most commonly used OC medium, which contains 2% fetal bovine serum (FBS), penicillin, streptomycin, and amphotericin B. The recommended storage time in OC is four weeks at 30°C-37°C. Ideally, the medium is renewed every one to two weeks, but some eye banks do not change the medium during storage time. However, the cornea inside OC becomes edematous, and one to seven days before grafting, the cornea is transferred from the storage medium into a dehydrating medium containing dextran at 20°C-35°C. Generally, a sample is taken for microbiologic testing from a storage medium every seven days or when it is renewed and from dehydrating medium as well [3].

In Saudi Arabia, all cases of corneal transplantation have been conducted utilizing imported donor tissues from the United States. All donor tissues were imported from Eye Bank Association of America (EBAA)-accredited centers and met EBAA minimum standards. All tissues were maintained in Optisol-GS storage media (Bausch & Lomb, Irvine, CA) inside expandable polystyrene shipping containers where they are shipped to Saudi Arabia through a non-stop flight within 13-24 hours inside a refrigerator at 4°C. Upon arrival, the containers are transferred to the eye bank at King Khalid Eye Specialist Hospital (KKESH) in Riyadh [4]. From there, donor corneal tissues are distributed and transported to many hospitals all over the country.

At DESH, we conducted a retrospective track of the corneal rim culture results of every patient who underwent any corneal transplantation procedure like penetrating keratoplasty (PKP), lamellar keratoplasty (LKP), Descemet's stripping automated endothelial keratoplasty (DSAEK), and Descemet's membrane endothelial keratoplasty (DMEK) between October 2016 and February 2020.

## Materials and methods

After obtaining approval from the Dhahran Eye Specialist Hospital (DESH) Institutional Review Board, results of corneal rim cultures between October 2016 and February 2020 were reviewed. A total of 684 corneal transplantation procedures were performed at DESH during this period. On the day of surgery, donor corneas were re-checked for suitability, then taken to the operating theater and kept in the operating room at 18°C-22°C, and trephined by the surgeon within 15 minutes. The remaining corneal rim was collected after trephination and sent for bacterial and fungal cultures.

The culture media used were anerobic blood agar (ANBA), chocolate agar (CHOC), and MacConkey agar (MAC). Sabouraud dextrose agar (SDA) was used for fungal culture. All cultures were preserved inside a VITEK Compact machine (BioMérieux, Marcy l'Etoile, France), which uses software to document the organism and antibiotic susceptibility. Gram and Giemsa stains were used to identify bacteria, whereas a potassium hydroxide (KOH) test was performed to identify fungi. Once there is growth in the culture media, the lab will inform the surgeon about the culture results for further possible management. The surgeon will report adverse reactions linked to donor tissue to the KKESH Eye Bank to inform the source eye bank. All cases then reported back to the EBAA.

On the first postoperative day, every patient was examined and discharged on moxifloxacin ophthalmic solution 0.5% antibiotic and prednisolone acetate ophthalmic suspension, USP 1%. A minority of patients who were known to have comorbidities were treated accordingly (e.g., antiglaucoma drops for glaucoma patients and systemic antiviral in herpetic eye disease). Patients were scheduled for follow-ups one day after surgery, then after one week, one month, three months, six months, nine months, 12 months, 18 months, and 24 months postoperatively.

## Results

Between October 2016 and February 2020, a total of 684 corneal transplantation procedures were performed at DESH. Routine donor rim cultures were done for all cases, and six cases were positive (0.88%). There were five positive fungal donor rim cultures (0.73%), and one corneal rim was mixed for both bacterial and fungal cultures (0.15%). Our results were consistent with the findings published in the literature in similar reports, which is 0.53%-15.7% [1].

The decision to start patients on medication in response to positive rim culture was made on an individual basis since there are no fixed protocols to decide the next step when a positive culture is discovered. The action was documented in response to positive cultures in two cases (33.33%). The type of the organism, sensitivity, clinical courses, and interventions are documented in Table 1.

**Table 1:** Patient information PKP: Penetrating keratoplasty; DALK: Deep anterior lamellar keratoplasty; DSAEK: Descemet stripping endothelial keratoplasty; KC: Keratoconus; VA: Visual acuity.

Patient number	#1	#2	#3	#4	#5	#6
Death to tissue preservation	11 hours	5 hours	10 hours	10 hours	11 hours	9 hours
Time from death to surgery	10 days	11 days	9 days	7 days	9 days	8 days
Type of surgery	DALK	PKP	PKP	PKP	DSAEK	DALK
Pre-op diagnosis	KC	KC	KC	KC	Decompensated cornea	KC
Resulting documentation time	4 days	11 days	4 days	5 days	7 days	5 days
Type of bacteria	-	-	Enterococcus fecalis	-	-	-
Bacterial antibiotic sensitivity	-	-	Levofloxacine, linezolide, and vancomycin	-	-	-
Type of fungi	Candida lusitaniae	Aspergillus	Candida albicans	Candida albicans	Candida glabrata	Candida glabrata
Fungal antibiotic sensitivity	Fluconazole, voriconazole, and caspofungin	Not mentioned	Fluconazole, voriconazole, and amphotericin B	Fluconazole and flucytosine	Amphotericin B and flucytosine	Amphotericin B
Clinical finding	Clear	Clear	Clear	Clear	Whitish plaques in the interface + Corneal decompensation + Hypopyon	Small wound dehiscence + well-defined whitish sheath-like opacity along the interface
Intervention	No intervention	No intervention	Voriconazole 1% QID for 3/12	No intervention	Amphotericin B 0.15%	No intervention
Outcomes	Clear graft	Clear graft	Clear graft	VA sc 20/100	AC wash + sub conj voriconazole (not responding)	Wound dehiscence re-suturing

Among these six patients, two patients (33.33%) who had a positive culture with candida required further interventions. None of these patients developed fungal endophthalmitis. Overall, there was a 0.29% incidence of fungal keratitis (2/684) in our study.

The first case who underwent DSAEK and had a positive culture with candida developed keratitis with hypopyon without endophthalmitis on a postoperative day 13 despite the use of specific antifungals in response to the positive culture. The patient required AC wash with intracameral voriconazole 0.05 mg/ml but was unsuccessful and eventually ended up with a therapeutic PKP.

The second case underwent DALK and also had a positive culture with candida. The primary surgeon preferred to follow the case closely and did not start antifungals as there was no sign of active infection. After six months, small wound dehiscence between two and three o'clock associated with well-defined whitish plaque opacity along the interface was documented. Re-suturing was done, but the condition deteriorated and eventually ended up with re-grafting.

## Discussion

The development of endophthalmitis post-keratoplasty is a vision-threatening complication with an incidence rate of 0.08%-0.77% [5,6]. To the best of our knowledge, this is the first study to report ocular infections post-keratoplasty with infected donor tissue storage in hypothermic medium (Optisol-GS) in Saudi Arabia.

The rate of the positive corneal rim cultures has been estimated to be 12%-39% for bacterial and 0%-12% for fungal despite the aseptic techniques and conditions used for preparing corneal grafts. Such methods include the use of 5% povidone-iodine solution, GS media, and storage in hypothermic conditions [7]. Receiving a culture-positive corneal donor rim was found to increase the risk of endophthalmitis by 12 times compared to those who received a cornea with a negative culture [5]. However, the predictability of ocular infection post-keratoplasty with a culture-positive donor rim remains a debatable subject in many studies, and for this discrepancy, the cost-effectiveness of routine corneal rime culture was a subject of controversy in the literature [8].

Over the last few years, an increase in fungal infective keratitis post-keratoplasty has been documented according to an EBAA medical report and many other publications [8-10]. The most common isolated fungus was Candida spp, while endothelial keratoplasty has demonstrated a higher affinity to acquire the infection than other forms of keratoplasty. The reasons why fungal infective keratitis surpasses those of bacterial infective keratitis can be attributed to the lack of antifungal agents in Optisol-GS media, the use of broad-spectrum antibiotics postoperatively, and an increased warming time used in endothelial keratoplasty preparation [10]. However, in Europe where corneas are preserved in OC and supplemented with amphotericin B, the rate of positive fungal corneal rim cultures and post-keratoplasty fungal infections is less [7].

Kiatos et al. in 2017 conducted a systematic review and cost-effectiveness study for corneoscleral rim cultures in keratoplasty and reported that 954 corneal rims out of 7,870 grafted cornea had positive corneal rim culture (12.1%), and 12 cases out of these 954 positive cultures developed ocular infections (1.3%). The estimated prevalence of ocular infections was 0.15%, 12 out of 7,870, where three cases had bacterial infections: two endophthalmitis and one keratitis, while nine cases had fungal infections: three cases had endophthalmitis and six cases had keratitis. They concluded that fungal rim cultures are cheaper, cost-effective, and have a positive predictive value compared to bacterial rim cultures [8].

Study limitations

The main limitations of this study were the small sample size, the relative rarity of ocular infections post-keratoplasty, and the retrospective data collection method that limits variable analysis.

## Conclusions

Our results concerning the outcomes of positive corneal rim cultures revealed that fungal contamination is more than bacterial contamination in corneas that are preserved in Optisol-GS media. These findings were within the same range reported in the literature. Despite the limitations of our study, we recommend obtaining routine corneoscleral rim cultures at the time of any keratoplastic surgery and following patients that develop positive donor fungal rim cultures in order to start appropriate treatment and prevent further progression of infection, and to avoid the high treatment burden of such cases.
